# Unlocking the potential of exercise: harnessing myokines to delay musculoskeletal aging and improve cognitive health

**DOI:** 10.3389/fphys.2024.1338875

**Published:** 2024-09-02

**Authors:** Xing Gao, Yiyan Chen, Peng Cheng

**Affiliations:** ^1^ Graduate School, Wuhan Sports University, Wuhan, China; ^2^ Department of Physical Education, Suzhou Vocational University, Suzhou, China; ^3^ Department of Basic Teaching, Suzhou City University, Suzhou, China

**Keywords:** exercise, musculoskeletal aging, mild cognitive impairment, Alzheimer’s disease, myokines, muscle-brain loop

## Abstract

**Objectives:**

This review aims to summarize the common physiological mechanisms associated with both mild cognitive impairment (MCI) and musculoskeletal aging while also examining the relevant literature on how exercise regulation influences the levels of shared myokines in these conditions.

**Methods:**

The literature search was conducted via databases such as PubMed (including MEDLINE), EMBASE, and the Cochrane Library of Systematic Reviews. The searches were limited to full-text articles published in English, with the most recent search conducted on 16 July 2024. The inclusion criteria for this review focused on the role of exercise and myokines in delaying musculoskeletal aging and enhancing cognitive health. The Newcastle‒Ottawa Scale (NOS) was utilized to assess the quality of nonrandomized studies, and only those studies with moderate to high quality scores, as per these criteria, were included in the final analysis. Data analysis was performed through narrative synthesis.

**Results:**

The primary outcome of this study was the evaluation of myokine expression, which included IL-6, IGF-1, BDNF, CTSB, irisin, and LIF. A total of 16 studies involving 633 older adults met the inclusion criteria. The current exercise modalities utilized in these studies primarily consisted of resistance training and moderate-to high-intensity cardiovascular exercise. The types of interventions included treadmill training, elastic band training, aquatic training, and Nordic walking training. The results indicated that both cardiovascular exercise and resistance exercise could delay musculoskeletal aging and enhance the cognitive functions of the brain. Additionally, different types and intensities of exercise exhibited varying effects on myokine expression.

**Conclusion:**

Current evidence suggests that exercise mediates the secretion of specific myokines, including IL-6, IGF-1, BDNF, CTSB, irisin, and LIF, which establish self-regulatory circuits between the brain and muscle. This interaction enhances cognitive function in the brain and improves skeletal muscle function. Future research should focus on elucidating the exact mechanisms that govern the release of myokines, the correlation between the intensity of exercise and the secretion of these myokines, and the distinct processes by which myokines influence the interaction between muscle and the brain.

## 1 Introduction

With a global increase in life expectancy, sarcopenia, cognitive impairment, frailty, and age-related anorexia, have become prominent issues in geriatric care (Morley, 2017b). A previous study reported that the global prevalence of dementia is expected to double every 20 years, potentially reaching 131.5 million patients by 2050 (Prince et al., 2015). A significant majority (60%–80%) of dementia cases comprise individuals with Alzheimer’s disease (AD), a neurodegenerative disorder. Mild cognitive impairment (MCI), on the other hand, serves as a transitional stage between mild dementia and normal aging, and often precedes the onset of AD (Grundman et al., 2004). Unlike the typical cognitive changes associated with aging, MCI often involves a decline in cognitive abilities, such as memory loss and learning difficulties, but does not meet the criteria for dementia. However, over 50% of individuals with MCI often progress to AD or other forms of dementia within 4–6 years (Hansson et al., 2006).

Sarcopenia and cognition, both of which are linked to aging, are also closely associated with each other and are crucial areas of ongoing research. Components of sarcopenia, such as gait speed and muscle strength, have been linked to cognitive impairment. Buchman et al. reported a 9% increase in the risk of AD for every 1-lb annual decrease in grip strength (Buchman et al., 2007). Additionally, gait speed is also a well-established predictor of dementia, especially in individuals with underlying cognitive impairment (Montero-Odasso et al., 2020). The EWGSOP2 criteria for muscle mass and gait speed correlate with a higher risk of mortality, indicating that a decrease in muscle mass or function is linked to an elevated risk of death (Cruz-Jentoft et al., 2019). Therefore, sarcopenia may lead to a strong bidirectional association between muscular dysfunction, diminished gait speed, and cognitive decline, which is often referred to as the muscle-brain loop.

Multiple studies have highlighted the importance of regular exercise in preventing and managing metabolic disorders, such as obesity, type 2 diabetes, and sarcopenia (Booth et al., 2012). Physical activity has also been linked to improved cognitive function and a reduced risk of neurodegenerative diseases (Tyndall et al., 2018). With chronic exposure to exercise, the body undergoes a series of adaptations to meet increased demands. This can involve changes in muscle strength, cardiovascular capacity, and metabolic efficiency, among other factors. The allostasis-interoception model posits that the brain plays a key role in predicting and meeting physiological needs resulting from regular exercise. By constantly monitoring internal conditions, the brain can adjust the body’s systems to maintain optimal function (Bobba-Alves et al., 2022). Regular physical activity plays a crucial role in maintaining the body’s ability to effectively anticipate and respond to stressors. Conversely, a sedentary lifestyle can disrupt this ability, leading to a decline in overall health and an increased risk of chronic conditions. Studies have indicated that sedentary behavior and a lack of physical activity are risk factors for sarcopenia in individuals with MCI and AD (Sugimoto et al., 2016), and consistent exercise or physical training can lead to various physiological changes that enhance skeletal muscle size and strength, ultimately aiding in counteracting the negative effects of sarcopenia in older adults. Despite these findings, a complete understanding of the molecular mechanisms involved is lacking.

A noteworthy contribution to this field is the concept of myokines (cytokines produced by muscles) by Pedersen and colleagues (Pedersen et al., 2003). Subsequently, Safdar and his team introduced the concept of exerkines, which include myokines as well as other compounds, such as metabolites, extracellular vesicles, and nucleic acids that are released during muscle contraction, all of which together form the myometabokiome (Safdar et al., 2016). The interactions among myokines, which are transported to various organs through the circulatory system, present a promising area of research as it may potentially offer insights into their access to the central nervous system (CNS). Therefore, in this research, we examined the molecular mechanisms underlying the role of exercise-regulated myokines in musculoskeletal aging and MCI, to provide new insights and identify therapeutic targets for the treatment of aging-associated skeletal muscle atrophy and MCI.

## 2 Methods

### 2.1 Search strategy and selection criteria

**FIGURE 1:**
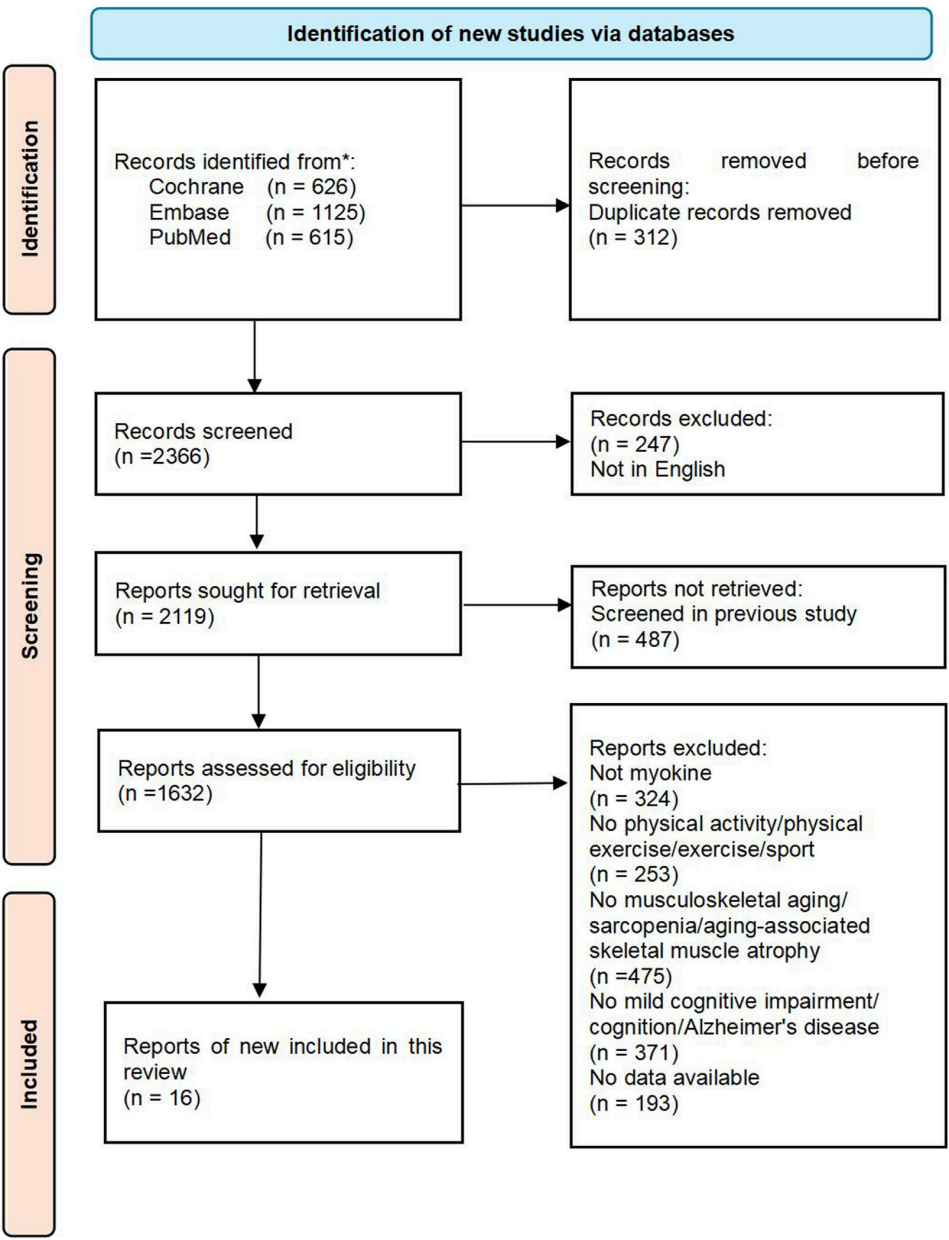
The flowchart of this review.

This review was conducted in accordance with the Preferred Reporting Items for Systematic Reviews and Meta-Analyses (PRISMA 2020) guidelines (Page et al., 2021). The literature search was carried out across various databases, such as PubMed (including MEDLINE), EMBASE, and the Cochrane Library of Systematic Reviews. The search was restricted to full-text articles published in English, with the most recent search conducted on 16 July 2024. The search terms used were “physical activity,” “physical exercise,” “exercise,” “sport,” “myokine,” “musculoskeletal aging,” “sarcopenia,” “aging-associated skeletal muscle atrophy,” “mild cognitive impairment,” “cognition,” and “Alzheimer’s disease”. Sixteen articles meeting the inclusion criteria were included after a thorough review process. The flowchart detailing the literature screening process can be found in Figure 1. Furthermore, a manual search of reference lists from review papers and articles in the final review was carried out. Three independent reviewers assessed papers for inclusion at three stages: title assessment, abstract assessment, and full paper assessment.

### 2.2 Selection criteria

The inclusion criteria for the studies focused on the role of exercise and myokines in delaying musculoskeletal aging and improving cognitive health. Studies had to be published in English language peer-reviewed journals and could include original research, randomized controlled trials, observational studies, cohort studies, and case‒control studies conducted on human subjects. The intervention had to involve exercise as the primary stimulus for myokine release, with reported quantitative outcomes related to musculoskeletal aging (e.g., muscle mass, strength, exercise performance) and/or mild cognitive impairment (e.g., cognitive function, memory). Attempts were made to contact the authors for additional information when possible. The exclusion criteria included animal and *in vitro* studies, as well as reviews; studies not directly focusing on the effects of exercise and myokines on musculoskeletal aging or cognitive health; studies with incomplete or unclear outcome reporting; and non-English language publications. Two reviewers independently screened titles, abstracts, and full texts of the articles for inclusion, with a third reviewer providing input in cases of disagreement.

### 2.3 Data extraction

Data extraction was performed systematically by two independent reviewers via a standardized data extraction form. The following information was extracted from each study: author(s) and publication year; study design (RCT, observational, cohort, case‒control); sample size and participant characteristics; exercise intervention details (type, intensity, duration, frequency); measured outcomes related to musculoskeletal aging and mild cognitive impairment; statistical methods used for analysis; and key findings and conclusions. Any discrepancies in data extraction were resolved through discussion between the reviewers or by consulting a third reviewer.

### 2.4 Quality assessment

The Newcastle‒Ottawa Scale (NOS) was utilized to evaluate the quality of nonrandomized studies (observational, cohort, case‒control) in three domains: selection of study groups, comparability of groups, and ascertainment of the outcome of interest. Only studies with moderate to high quality scores, as determined by these criteria, were included in the final analysis.

### 2.5 Data analysis

Data analysis was conducted via narrative synthesis. The narrative synthesis involved summarizing the findings of each study and identifying common themes and patterns across studies. The results of the review were presented in a nonstructured format, including the main advancements in this topic.

## 3 Result

### 3.1 Study characteristics

The section detailing the study characteristics highlights the essential components of the systematic review methodology and the studies included, in accordance with PRISMA guidelines. Illustrated in Figure 1, the flowchart demonstrates the meticulous selection procedure employed to identify and screen relevant studies. This review incorporated a comprehensive search strategy designed to encompass all pertinent investigations assessing the potential of exercise to leverage myokines for delaying musculoskeletal aging and enhancing cognitive health. The search spanned several electronic databases without restrictions regarding publication dates or languages, thereby ensuring a diverse and up-to-date array of evidence. Musculoskeletal aging is defined as a process characterized by a reduction in skeletal muscle mass and functionality, typically resulting from the natural aging process and a decline in physical activity levels.

In addition, the studies included in this review had different designs, including randomized controlled trials (RCTs), observational studies, cohort studies, and case-control studies. To ensure the robustness of study results, NOS rigorously evaluates the study quality of non-randomized studies. Specifically, NOS is used to assess study group selection and comparability between groups and to identify exposures or outcomes in cohort and case-control studies. It is worth noting that the NOS score of all studies was 7 out of 9, indicating that the included retrospective cohort studies had acceptable bias. Furthermore, most of the studies included in this review were preclinical studies designed to investigate the role of myokines in delaying and ameliorating musculoskeletal aging. The studies are summarized in Table 1. This review includes sixteen studies, from which data extraction was systematically conducted by two independent reviewers using a standardized form. This approach was implemented to minimize errors and ensure consistency. Key information extracted from each study encompassed the author(s) and publication year, study design, sample size, participant characteristics, details of the exercise intervention (including type, intensity, duration, and frequency), outcomes measured in relation to musculoskeletal aging and mild cognitive impairment, and the statistical methods utilized for analysis. This rigorous methodology in data extraction and analysis ensures that the findings of the review are based on a thorough and precise synthesis of the available evidence. In summary, this review offers a comprehensive and reliable synthesis of the existing evidence regarding the potential of exercise to utilize myokines in mitigating musculoskeletal aging and enhancing cognitive health.

**TABLE 1:** Effect of different exercise modes on musculoskeletal aging and mild cognitive impairment.

Myokine	Model	Sample size	Exercise mode	Exercise duration and frequency	Change in myokine levels	Source of myokine	References
IL-6	69.9 ± 7.5 years old elderly with AD	198 participants	Treadmill/Stationary bike/Cross trainer exercise	Moderate-to-high intensity 60 min, 12 weeks	IL-6↑	Plasma CSF	Jensen et al. (2019)
IL-6/Irisin	67 ± 8 years old elderly women	27 participants	Nordic walking training	60%–70% intensity of walking 60 min, 12 weeks	IL-6↓ irisin↑	Plasma	Gmiat et al. (2017)
IL-6	≥60 years elderly	13 participants	Otago exercise program	3 times/week, 30 min 8 weeks	IL-6↓	Serum	Gde Agung Mahendra et al. (2022)
IL-6	>65 years elderly	81 participants	Strengthening exercise	3 times/week, 50 min 3 months	IL-6↓	Serum	Heo and Jee (2024)
IL-6/Irisin/LIF	68 ± 5 years elderly	7 participants	Blood-flow resistance exercise	40%–60% of 1 RM, 10x 60%–80% of 1 RM, 3–5x	IL-6, LIF↓ irisin↑	Plasma	Cordingley et al. (2023)
IL-6	≥65 years elderly	7 participants	Single resistance exercise	60%–80% of 1RM; 8–12x	IL-6 -	Plasma	Cornish et al. (2018)
IGF-1	>70 years elderly	3 participants	Resistance training	10-week	IGF-1↑	Skeletal muscle	Urso et al. (2005)
IGF-1	65–92 years old women	41 participants	Elastic band resistance training	60 min, 2 times/week 6 months	IGF-1-	Serum	Hofmann et al. (2016)
IGF-1	69.9 ± 5 years old women MCI	75 participants	Progressive resistance strength training	24 months	IGF-1↑	Plasma	Molina-Sotomayor et al. (2020)
IGF-1/IL-6	60–85 years old probable MCI	52 participants	Progressive resistance exercise program for the lower limbs	70%–75% of 1 RM; 8–10x, 1–3 week 75%–80% of 1 RM; 6–8x, 4–9 week 80%–85% of 1 RM; 6 x, 10–12 week	IGF-1 IL-6↑	Serum	Vints et al. (2024)
IGF-1/BDNF	60–80 years old aMCI	46 participants	Acute cardiovascular/Resistance exercise	One-time	IGF-1 BDNF↑	Plasma	Tsai et al. (2018a)
IGF-1/BDNF	>65 years elderly women	22 participants	Aquatic exercises	60 min, 3 times/week 12 weeks	IGF-1 BDNF↑	Plasma	Kang et al. (2020)
IGF-1/BDNF	>60 years elderly men	20 participants	Resistance exercise/Running	65%–70% of 1 RM, 10x 65%–70% of HR max, 30 min	IGF-1 BDNF↑	Plasma	Arazi et al. (2021)
BDNF	65–92 years old MCI	23 participants	High-intensity cardiovascular exercise	75%–85% of HRmax, 45–60 min 4 times/week, 6 months	BDNF↓	Plasma	Baker et al. (2010)
Irisin	>65 years elderly	7 participants	Elastic band exercise	2 times/week, 12 months	irisin↑	Serum	Kim et al. (2015)
CTSB	= 64.9 years old late middle-aged adults	11 participants	Moderate to-vigorous intensity cardiovascular exercise	150 min/week; 26 weeks	CTSB↑	Plasma	Gaitán et al. (2021)

RM, repetition maximum; HRmax, maximal heart rate; X, repetition; MCI, mild cognitive impairment; aMCI, amnestic mild cognitive impairment; CSF, 160 cerebrospinal fluid; VO2 max, maximum oxygen consumption.

## 4 Discussion

### 4.1 Musculoskeletal aging is an independent risk factor for MCI, and *vice versa*

Meta-analyses of the studies published over the past decade have revealed a consistent two-fold increase in the likelihood of cognitive impairment among older adults with sarcopenia compared to those without this condition (Peng et al., 2020). Sarcopenia and cognitive impairment are significant contributors to disability in elderly individuals. For instance, in patients with AD, the prevalence of sarcopenia is considerably high even in the early stages of the disease. Moreover, advanced age, a reduced BMI, and a decreased Mini-Mental State Examination (MMSE) score are also linked to sarcopenia in both male and female patients with AD (Ogawa et al., 2018). One cross-sectional survey confirmed a positive correlation between sarcopenia and MCI in low- and middle-income countries (LMICs) (Jacob et al., 2021). Another previous study indicated that the scores on the Short Physical Performance Battery (SPPB) correlate positively with the risk of cognitive impairment in older individuals (Moon J. H. et al., 2016). Additionally, a slow gait has also been suggested as an early indicator of cognitive impairment and the onset of dementia (Verghese et al., 2007; Mielke et al., 2013). According to one study, cognitive impairment is linked to sarcopenia primarily due to its correlation with a slow gait. Cognitive impairments in elderly men, including issues with processing speed and executive functions, are connected to sarcopenia, as well as a slow and weak gait (Kim and Won, 2019). These findings suggest a strong relationship between sarcopenia and the cognitive impairment associated with AD and dementia, thereby highlighting the potential role of sarcopenia in the pathophysiology of these disorders.

Sarcopenia and cognitive impairment share certain common risk factors, such as cerebrovascular disease, diabetes, and hypertension. Studies have shown that following a Mediterranean diet rich in high-quality protein may reduce the risk of developing MCI (Wu et al., 2019). However, a lack of physical activity due to a sedentary lifestyle and a lack of exercise in patients with sarcopenia can further reduce cognitive function and stability in these patients (Witham et al., 2020). In summary, sarcopenia is a potential risk factor for cognitive impairment and a potential predictor of the likelihood of cognitive impairment. However, further investigations are warranted to identify the causal association between sarcopenia and MCI.

### 4.2 Musculoskeletal aging and MCI: overlapping risk factors and potential mechanisms

**FIGURE 2:**
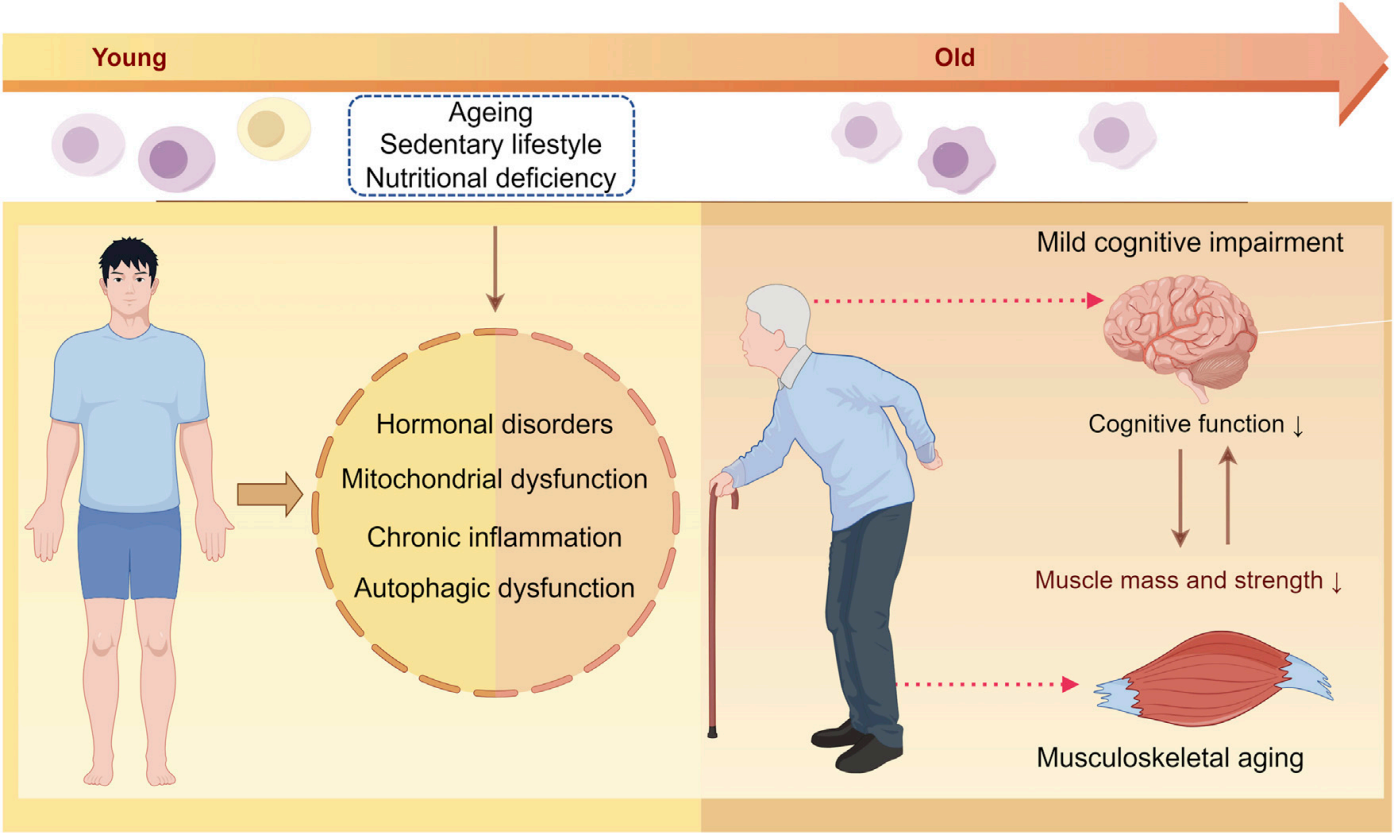
Illustration of the potential mechanisms underlying musculoskeletal aging and mild cognitive impairment. With aging, factors such as a sedentary lifestyle, insufficient physical activity, and nutritional deficiencies can lead to hormonal imbalances, mitochondrial dysfunction, chronic inflammation, and impaired autophagy in both the skeletal muscles and brain tissues. These changes ultimately affect the maintenance of the skeletal muscle mass and impact cognitive function.

Skeletal muscle atrophy and cognitive impairment are frequently observed in aging individuals, suggesting a potential connection between the two. While the negative impacts of muscle loss on daily functioning and wellbeing are well documented, the specific biological pathways linking sarcopenia and cognitive decline are not fully understood. However, the potential physiological mechanisms common between the two include nutritional deficiencies, hormonal imbalances, mitochondrial dysfunction, chronic inflammation, and irregularities in autophagy (Figure 2).

#### 4.2.1 Hormonal disorders

The dysregulation of endocrine hormones, such as insulin-like growth factor (IGF), growth hormone (GH), testosterone, estrogen, and cortisol, during aging is closely associated with sarcopenia and cognitive impairment (Zeng et al., 2020; Dabin et al., 2022). A decrease in IGF expression with advancing age leads to limited IGF/PI3K/Akt signaling-mediated protein synthesis, a reduction in the number of satellite cells available for damage repair, and ultimately, a decrease in muscle mass and strength (Serra et al., 2007). IGF-1 plays a crucial role in preventing cell death, promoting neurogenesis in the hippocampus, facilitating the normal phosphorylation of tau proteins, and clearing Aβ. Therefore, reduced IGF-1 levels in the bloodstream of patients with AD have been linked to increased Aβ deposition in the brain of these individuals (Westwood et al., 2014). GH, on the other hand, promotes muscle growth directly via GHR or indirectly by stimulating IGF-1 production. In the brain, GH activation in the hypothalamus triggers the release of GH-releasing hormone (GHRH), leading to elevated circulating levels of IGF-1, thereby supporting neuronal growth and survival, regulating tau protein phosphorylation, and counteracting the negative effects of Aβ *in vivo* (Winston et al., 2018). Furthermore, in the context of aging, a decrease in the level of testosterone, a critical sex hormone, has been associated with a decrease in muscle mass and strength (Mouser et al., 2016). Testosterone also plays a role in inhibiting SMAD-mediated protein degradation through the ubiquitin-proteasome system, thus reducing protein atrophy. While age-related decreases in testosterone levels appear to increase disease risk in humans, estrogen activates dormant muscle satellite cells, aiding in the repair of damaged muscle tissues (Morley, 2017a; Liao et al., 2019). However, compared to testosterone, estrogen, which possesses potential neuroprotective functions, appears to have a reduced ability to protect microglia from Aβ toxicity and promote microglial Aβ clearance (Keyvani et al., 2018).

In addition to anabolic hormones, glucocorticoids, particularly cortisol, also play a significant role in influencing human skeletal muscles (Kaplan and Shimizu, 1963). Cortisol levels are regulated at both the systemic and tissue levels to maintain glucocorticoid homeostasis. Elevated cortisol levels can result in muscle atrophy by increasing proteolysis through the ubiquitin-proteasome and lysosome systems (Sato et al., 2017). Additionally, glucocorticoids can decrease muscle protein synthesis by inhibiting IGF-I signaling and increasing myostatin signaling. This dual effect of cortisol on the muscles can impact muscle function and overall muscle health (Schakman et al., 2013). Furthermore, studies have revealed a correlation between plasma cortisol levels and cognitive impairment in individuals with AD, suggesting that the hyperactivity of the hypothalamic-pituitary-adrenal axis, which leads to elevated cortisol levels, may contribute to the progression of cognitive decline in these individuals (Zvěřová et al., 2013; Popp et al., 2015). Moreover, cortisol levels tend to increase with age, and higher levels of cortisol are associated with poorer cognitive function (Roelfsema et al., 2017; Qiu et al., 2021). These findings highlight the importance of understanding the impact of hormone levels on both muscle health and cognitive function, especially in the context of aging and neurodegenerative diseases.

#### 4.2.2 Mitochondrial dysfunction

The increased production of reactive oxygen species (ROS) during aging is associated with increased damage to biomolecules (Radak et al., 2016). This increase in oxidative damage results in the development of different pathophysiological states, such as aging-related skeletal muscle loss and neurodegenerative disorders (Naudí et al., 2007; Tang et al., 2019). Additionally, the decrease in antioxidant activity with aging leads to the activation of nuclear factor kappa-B (NF-κB) and forkhead box O (FoxO) through the ROS pathways, thus triggering apoptosis and protein degradation in skeletal muscles. These transcription factors subsequently promote the expression of the muscle atrophy F-box protein (MAFbx/Atrogin-1) and the muscle-specific ring finger protein 1 (MuRF1), ultimately resulting in skeletal muscle atrophy.

Compromised mitochondrial function leads to the expression of oxidative stress markers, such as IL-6, CRP, and IL-1RA, which are closely associated with reduced physical function and muscle strength in elderly individuals (Bellanti et al., 2018). Therefore, the maintenance of mitochondrial health is crucial for the viability and functioning of neurons, as their structural and functional integrity are closely intertwined (Rugarli and Langer, 2012). Moreover, the maintenance of normal mitochondrial function is essential for maintaining the stability of the internal environment in mammals, and any disruption in the ROS scavenging system during mitochondrial oxidative phosphorylation can lead to brain aging and neurodegenerative disorders (Bernardo et al., 2013). Recent studies have highlighted a strong relationship between oxidative stress, aging, and neurodegenerative conditions. These findings suggest that the accumulation of Aβ triggers detrimental cellular changes, including oxidative damage to the mitochondria (Bozner et al., 1997; Pappolla et al., 1998). However, the precise mechanisms by which Aβ operates *in vivo* are not fully understood. Some studies have proposed that the translocase of the outer mitochondrial membrane 40 (TOMM40) plays a role in regulating the entry of Aβ into the mitochondria through the Tom40 outer membrane pore, underscoring its role as a potential target for the treatment of cognitive impairment (Yu et al., 2007). Collectively, these reports suggest that the restoration of mitochondrial function is crucial for the effective management of nerve disorders, especially cognitive dysfunction.

#### 4.2.3 Chronic inflammation

Aging is commonly associated with a chronic low-grade inflammation known as inflammaging, which results in an elevation in the levels of inflammatory factors, which in turn cause the heightened metabolic degradation of skeletal muscles (Sato et al., 2016). Multiple potential mechanisms underlie chronic low-grade inflammation and its negative impacts on muscle health during the aging process. Age-related redox imbalances can lead to the upregulation of pro-inflammatory cytokines, which are key players in inflammatory pathways. Additionally, ROS can trigger inflammation, causing the release of TNF-α, leptin, and GH, and this cascade ultimately results in insulin resistance, accelerated muscle breakdown, and the loss of muscle mass (Thoma and Lightfoot, 2018). Proinflammatory cytokines, on the other hand, can counteract the anabolic effects of GH and IGF-1, causing a negative muscle protein balance, thereby affecting muscle function and strength (Kim et al., 2018). Recent evidence suggests that age-related chronic inflammation is correlated with elevated levels of circulating biomarkers, such as TNF-alpha, IL-1beta, IL-6, COX-2, iNOS (Chung et al., 2009). The findings reveal that this inflammatory response contributes to increased muscle breakdown and depletion through various pathophysiological pathways, thus contributing significantly to the aging of skeletal muscles (Kedlian et al., 2024). TNF-α, another key cytokine, has also been implicated in chronic inflammation and the onset of neurodegeneration (Hofman et al., 1989; Mogi et al., 1994). Furthermore, inflammatory cytokines have been shown to interfere with insulin function at the cellular level and worsen its neurotoxic effects, thus exacerbating cognitive decline (Fava et al., 2017). Therefore, chronic inflammation may serve as a shared pathogenic factor for aging-associated muscular dystrophy as well as MCI.

#### 4.2.4 Nutritional deficiency

Aging-associated muscular dystrophy is a distinct disease associated with malnutrition. Reduced dietary intake and protein synthesis in patients with cognitive impairment can result in muscle loss, exacerbating the development of skeletal muscle atrophy (Cederholm et al., 2017). Malnutrition in elderly individuals, on the other hand, can result in decreased physical fitness and muscle strength and fitness, thus creating a vicious cycle. Inadequate nutritional intake can downregulate the Akt/mTORC1 signaling pathway, thus reducing muscle protein synthesis by inhibiting the phosphorylation of the downstream markers p70S6K and rpS6, and ultimately resulting in sarcopenia (Margolis et al., 2016). Inadequate nutrient intake can also increase oxidative stress or inhibit antioxidant mechanisms, contributing to the onset and progress of dementia. Evidence suggests that decreased food intake and activity in elderly individuals can cause vitamin D deficiency, which in turn impacts muscle protein synthesis and its metabolism through disturbances in the calcium and phosphorus levels and via insulin imbalance, ultimately leading to the development of sarcopenia (Ceglia et al., 2010). Moreover, since vitamin D and physical activity act synergistically and promote muscle protein ubiquitination and degradation, physical inactivity and vitamin D deficiency can worsen muscle atrophy (Yang et al., 2020). Furthermore, the risk of cognitive impairment is also greater in individuals with vitamin D deficiency, as vitamin D regulates calcium balance and Aβ deposition and displays antioxidant and anti-inflammatory properties in patients with AD.

#### 4.2.5 Autophagic dysfunction

Autophagy plays a crucial role in breaking down dysfunctional organelles and damaged macromolecules in aging cells, thus aiding in the maintenance of the internal environment of the muscle cells. Evidence suggests that the inhibition of autophagy in skeletal muscles can lead to neuromuscular synaptic issues and reduced muscle strength, potentially impacting the quality of life and the lifespan of animals (Carnio et al., 2014). This close association between autophagy and aging results in reduced autophagy or disrupted autophagic flux, leading to the excessive degradation of muscle proteins, thus exacerbating skeletal muscle loss. The age-associated reduction in neuronal autophagy, on the other hand, hinders mitochondrial renewal, causing the accumulation of impaired mitochondria, accelerated cell death, and inflammation (Sheng et al., 2010). Mounting evidence indicates that the interplay between cellular autophagy and mitochondrial dysfunction can contribute to the onset of various age-associated disorders (Green et al., 2011).

Similar to replicating cells, neurons, which are terminally differentiated post-mitotic cells during early development, do not possess the ability to eliminate misfolded proteins and damaged organelles through cell division (Metaxakis et al., 2018). Therefore, the dysfunction of the autophagic lysosomal degradation process disrupts neuroendocrine homeostasis, causes lipofuscin accumulation, inhibits autophagy, and establishes a detrimental cycle. Evidence suggests that the accumulation of autophagosomes in the hippocampus of aged mice due to reduced degradation is possibly linked with cognitive impairment (Soontornniyomkij et al., 2012). In addition, brain aging is also characterized by a gradual decline in neuronal autophagic lysosome function, which plays a crucial role in the progression from normal aging to pathological aging, ultimately resulting in neurodegeneration in patients with AD (Ling and Salvaterra, 2011). These findings suggest that impaired autophagy can contribute significantly to cognitive decline in elderly individuals.

### 4.3 Crosstalk between the muscles and the brain in musculoskeletal aging and MCI

**FIGURE 3:**
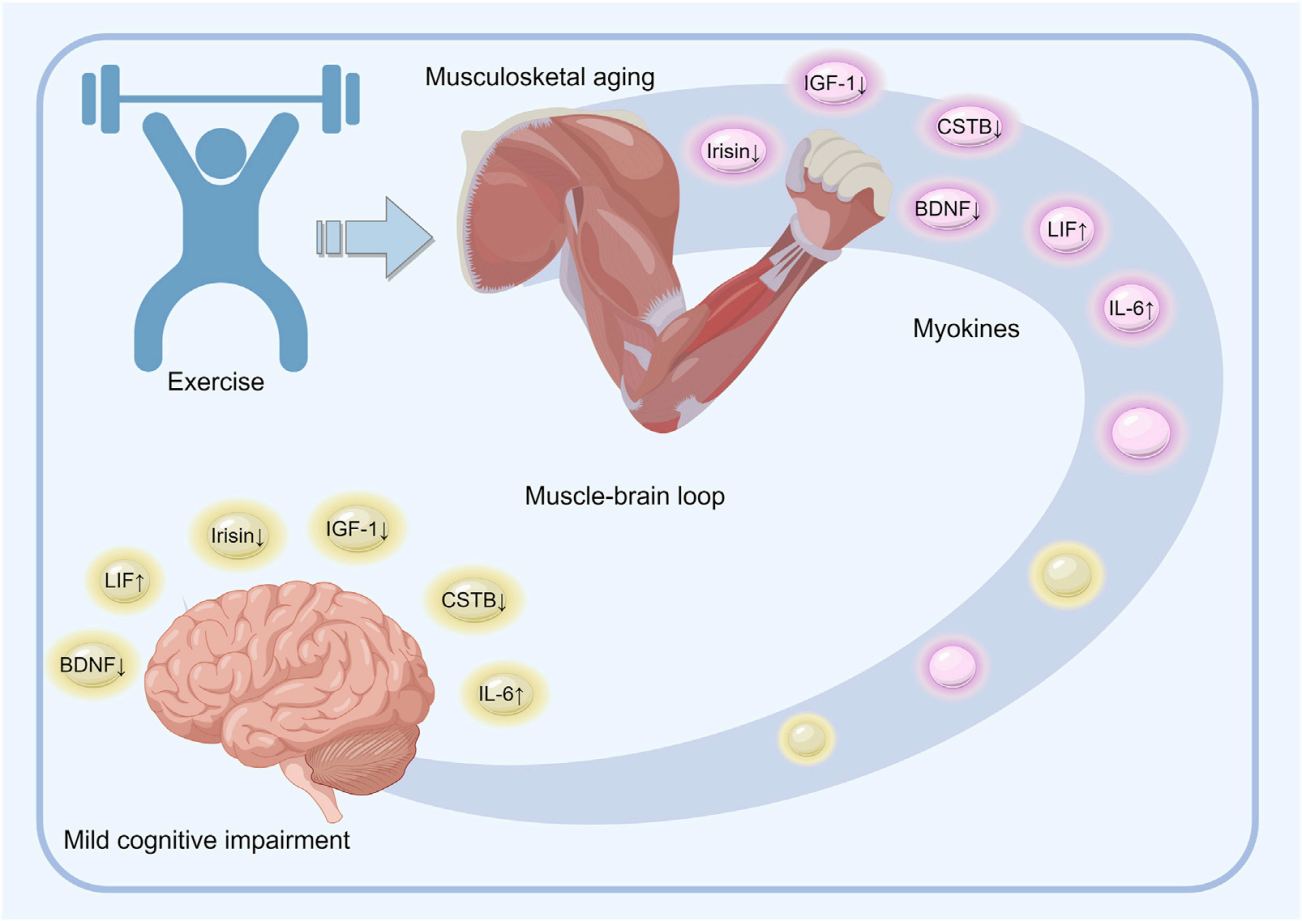
Overview of the mechanisms underlying the role of exercise-regulated myokines in musculoskeletal aging and mild cognitive impairment. Physical activity induces the release of various muscle-derived factors, such as IL-6, IGF-1, BDNF, CTSB, irisin, and LIF, into the bloodstream during muscle contraction. These factors either circulate freely or are enclosed in small vesicles that are capable of crossing the blood-brain barrier and impacting the cognitive and executive brain regions. The brain, on the other hand, modulates muscle activity and peripheral metabolism by directly influencing the target tissues and regulating cortisol levels through the hypothalamic–pituitary–adrenal axis. Effective coordination between the brain and muscles is thus essential for maintaining brain function, preventing cognitive decline, and preserving muscle mass.

Exercise stimulates the production and autocrine secretion of myokines, which regulate muscle health (Boström et al., 2012; Eaton et al., 2018; Cai et al., 2019). With age and decreased physical activity, there is a decline in skeletal muscle mass, which leads to reduced myokine secretion and a detrimental cycle. Conversely, physical activity has a neuroprotective effect, counteracting the cognitive decline caused by neurodegenerative processes. Notably, physical activity may have fewer side effects compared to the therapies currently in use for neurodegenerative disorders. The myokines released during physical activity can improve cognitive function, memory, neuroplasticity, appetite, and mood, and reduce neuroinflammation through peripheral mechanisms. IL-6, IGF-1, BDNF, CTSB, irisin, and LIF are some of the key myokines that jointly participate in the regulation of musculoskeletal aging and MCI (Figure 3).

#### 4.3.1 IL-6

Prolonged exercise leads to the production and release of IL-6, which is classified as a myokine, by the skeletal muscles**.** However, the release of IL-6 during exercise is associated with muscle damage (Bruunsgaard et al., 1997), and increased levels of circulating IL-6 have been linked to mortality in healthy elderly individuals (Harris et al., 1999). In a previous study, individuals with elevated levels of both IL-6 and CRP exhibited increased fibrinogen levels, indicating the potential presence of a chronic low-level inflammation associated with aging (Cohen et al., 1997). While elevated levels of IL-6 in the plasma lead to skeletal muscle atrophy through the STAT3/5 signaling pathway, the inhibition of the IL-6 receptor reduces muscle atrophy by suppressing the expression of MuRF1 (Yakabe et al., 2018). Therefore, IL-6 not only impedes muscle growth and disrupts energy balance but also actively contributes to muscle breakdown and subsequent atrophy (Goodman, 1994; Tsujinaka et al., 1995). Studies on transgenic mice with increased expression levels of human IL-6 have demonstrated significant muscle wasting in the mice by 10 weeks of age, along with the activation of lysosomal enzymes and increased expression of proteasomal subunits (Tsujinaka et al., 1995; Tsujinaka et al., 1996). However, the inhibition of IL-6 signaling by the prolonged administration of an IL-6R antibody led to the reversal of the muscular alterations observed in the IL-6 transgenic mice, and the alleviation of muscle atrophy in wild-type mice with tumors. Various studies have indicated that the Otago exercise program (Gde Agung Mahendra et al., 2022) and moderate-intensity strengthening resistance exercise (Heo and Jee, 2024) effectively decrease the risk of falls among elderly individuals by increasing muscle strength and stability and lowering the levels of IL-6.

IL-6, a key player in the early stages of amyloid plaque formation in the brain of patients with AD (Huell et al., 1995), has also been linked to tau phosphorylation, synapse loss, and cognitive impairment in mouse models of AD (Quintanilla et al., 2004). Despite certain conflicting opinions from the academic community, previous meta-analyses have shown that IL-6 levels are elevated in both the cerebrospinal fluid (CSF) and the plasma of individuals with MCI and AD compared to that in the CSF and plasma of control subjects (Brosseron et al., 2014). In individuals with AD, the elevation in the levels of IL-6 in both the brain and the bloodstream is associated with the severity of dementia (Huell et al., 1995; Kálmán et al., 1997). However, recent research has shown that the neutralization of IL-6 and the inhibition of the STAT3 signaling pathway in the brain of AD mouse models can improve memory impairment (Lyra et al., 2021). Despite its proinflammatory properties, IL-6 displays different effects during physiological and pathological conditions. One study revealed that the positive effects of Nordic walking are manifested by a decreased concentration of proinflammatory proteins such as HMGB1 and IL-6 and increased irisin (Gmiat et al., 2017). IL-6 can be considered both a pro-inflammatory protein and an anti-inflammatory protein. Previous research has reported significant variations in IL-6 levels in response to exercise, which are influenced by factors such as intensity, duration, working muscle mass, and an individual’s endurance capacity (Febbraio and Pedersen, 2005; Petersen and Pedersen, 2005). Overall, regular exercise has been associated with a significant reduction in inflammation by decreasing the circulating levels of specific inflammatory markers, including IL-6 (Bautmans et al., 2021).

#### 4.3.2 IGF-1

IGF-1 plays a crucial role in promoting mesenchymal stem cell differentiation and migration, as well as enhancing muscle fiber proliferation, differentiation, and contraction. IGF-1 also contributes to nerve remodeling and skeletal muscle regeneration by acting as a powerful neurotrophic factor that supports nerve regeneration and facilitates neuromuscular repair (Apel et al., 2010). The IGF-1/Akt/mTOR signaling pathway is known to regulate skeletal muscle hypertrophy (Rommel et al., 2001), and higher levels of IGF-1 can inhibit the expression of the protein degradation markers Atrogin-1 and MuRF1, thereby alleviating skeletal muscle atrophy (Schulze et al., 2005). Nevertheless, research indicates that this age-related decrease in IGF-1 levels in the skeletal muscles can be partially counteracted by progressive resistance training (Urso et al., 2005).

In addition to its role in the growth and development of the body, IGF-1 plays a key role in the development and metabolism of the CNS. A recent study in male mice has shown that the overexpression of IGF-1 in the CNS can protect against age-related cognitive dysfunction (Farias Quipildor et al., 2019)**.** For example, in a rat model of sporadic Alzheimer’s disease (sAD), adenovirus-mediated IGF-1 gene transfer into the brain led to an increase in the levels of synaptic markers, leading to enhanced hippocampal synaptic plasticity, thereby improving recognition and spatial memory (Zappa Villar et al., 2021). In humans, on the other hand, IGF-1 deficiency results in cognitive dysfunction, which can be improved by interventions that increase circulating IGF-1 levels (Sonntag et al., 2013). Additionally, exercise has also been found to counteract the negative effects of MCI by regulating the serum IGF-1 levels (Tsai C. L. et al., 2018). In aging rats, an 8-week swimming training program led to the activation of the IGF-1/PI3K/Akt signaling pathway in the hippocampus, resulting in the reduced apoptosis of the hippocampal neurons and improved hippocampal function (Lin et al., 2020). In humans, while studies on older women have shown that 16 weeks of aquatic exercise and 24 months of progressive resistance exercise can increase serum IGF-1 levels (Kang et al., 2020; Molina-Sotomayor et al., 2020), a study on 30 elderly males demonstrated that strength and endurance training can elevate the serum IGF-1 levels and enhance cognitive function in elderly individuals (Arazi et al., 2021).

#### 4.3.3 BDNF

BDNF, a protein belonging to the neurotrophic family, is primarily found in the brain and skeletal muscles and is vital for the development and differentiation of myoblasts and myofibers. It has also been detected in various tissues and body parts, including the periphery and the CNS, suggesting the presence of a neurotrophic connection between the different systems of the body and the blood-brain barrier (BBB) (Ribeiro et al., 2021). The level of BDNF decreases during myoblast maturation and myogenic differentiation, thus impacting the satellite cells or muscle progenitor cells (MPCs) and supporting the early differentiation of myoblasts (Mousavi and Jasmin, 2006). A study on a murine injury model revealed that BDNF functions via the tropomyosin-related kinase-B receptor (TrkBR) and the p75 neurotrophin receptor (p75NTR), both of which are markers of highly differentiated muscle precursor cells, thus enhancing myogenesis and myofiber maturation. Moreover, the expression of BDNF in the muscle satellite cells is upregulated during muscle injury, thereby facilitating the activation of the satellite cells (Yarrow et al., 2010).

BDNF also plays a crucial role in the CNS, overseeing neuronal survival, growth, and maintenance. It also regulates synaptic plasticity, cell survival, and brain cell differentiation (Aguado et al., 2003). Studies in mice have shown that BDNF positively affects brain function by promoting hippocampal cell regeneration, increasing *BDNF* gene expression, enhancing spatial memory, improving motor performance, and preserving the overall functionality of the brain (Marlatt et al., 2012). BDNF also acts as a key factor in exercise-induced neuroprotection, mediating the changes in synaptic strength and enhancing neurotransmitter release. Even in case of advanced memory impairment, cardiovascular exercise has been found to elevate BDNF levels, which in turn enhance hippocampal neuroplasticity and memory function in mice across various age groups from young to old (Tsai S. F. et al., 2018). Both human and animal studies have shown that acute/long-term cardiovascular/resistant exercise elevates circulating BDNF levels. In women, high-intensity cardiovascular exercise results in increased insulin sensitivity and reduced circulating levels of cortisol and BDNF, along with cognition-enhancing effects that are most pronounced for executive control tasks (Baker et al., 2010). In aging rats, cardiovascular exercise and strength training improve spatial memory by inducing neuroplasticity through distinct molecular mechanisms. Both exercise protocols also led to an increase in BDNF levels after training. While cardiovascular exercise specifically increased the levels of glutamatergic proteins (the NMDA receptor and PSD-95), strength training increased the levels of PKCα and the pro-inflammatory factors TNF-α and IL-1β (Vilela et al., 2017). These results suggest that cardiovascular exercise can positively impact brain functionality by affecting BDNF levels.

#### 4.3.4 Irisin

A study from 2012 revealed that prolonged exercise training in mice led to a significant increase in the expression of PGC-1α in the muscles, which in turn led to the enhanced expression of the target gene, *FNDC5*, resulting in the production of irisin, a myokine that converts white adipose tissue to brown fat (Boström et al., 2012). Irisin is a novel cytokine that is primarily found in the brain and skeletal muscles. Although its secretion decreases with age, it can be partially restored by resistance exercise training (Kim et al., 2015). Generally, individuals with sarcopenia exhibit lower levels of skeletal muscle protein biomarkers, including irisin, compared to those without sarcopenia (Yen et al., 2022). Irisin, an exercise-induced polypeptide secreted by skeletal muscles, crosses the blood-brain barrier and brings about certain exercise-mediated effects in the brain. Previous studies have shown a positive correlation between irisin levels and aging-induced cognitive dysfunction^.^ The primary pathological process of AD involves the over-accumulation of Aβ in the brain due to an imbalance in Aβ production and clearance (Lourenco et al., 2019). This excessive accumulation of Aβ leads to neurotoxicity, resulting in a decrease in the number of neurons, loss of synapses, and impaired nerve signal conduction, ultimately leading to cognitive impairment. Notably, FNDC5, the irisin precursor, has been found in various regions of the brain and is associated with neural differentiation (Hashemi et al., 2013). Physical exercise has been shown to enhance *FNDC5* expression (Wrann et al., 2013) and irisin levels (Siteneski et al., 2018) in the hippocampus. In a study involving rats, the administration of Aβ1‒42 to the hippocampus led to spatial learning and memory impairments, along with reduced hippocampal FNDC5 expression. However, 4 weeks of moderate-intensity running exercise increased the hippocampal FNDC5 mRNA levels and improved spatial learning and memory loss in the rats.

#### 4.3.5 Cathepsin B

Cathepsin B (CTSB), which is expressed in various tissues, including the skeletal muscles and the brain, can play a crucial role in brain flexibility. Moon et al. identified CTSB as a myokine in a study utilizing rat myotubes treated with the AMPK agonist AICAR (Moon H. Y. et al., 2016). Typically, physical activity increases the CTSB level in the blood and the *CTSB* gene expression in the hippocampus, indicating that CTSB impacts brain function both directly and indirectly. In mice, the deletion of the *CTSB* gene prevented the exercise-induced improvement in spatial memory retention and adult neurogenesis, reduced the inhibitory signals transmitted to dentate granule cells (GCs), and reduced the levels of hippocampal P11, a crucial protein necessary for the effect of CTSB on the differentiation and migration of neurons (Moon H. Y. et al., 2016). Additionally, CTSB also possessed the ability to penetrate the blood-brain barrier. While CTSB administration did not affect hippocampal cell proliferation in the mice, it increased the BDNF mRNA and protein expression levels. Additionally, CTSB treatment also elevated the level of doublecortin, a protein known for its neuroprotective effect and ability to promote neuronal migration.

CTSB is a potential therapeutic target for neurodegenerative diseases such as AD. Plasma levels of CTSB increased after adults at high risk for AD participated in 26 weeks of moderate-to-vigorous intensity cardiovascular exercise, with this alteration in CTSB positively correlated with cognitive performance (Gaitán et al., 2021). In a mouse model of AD, CTSB was found to reduce the levels of Aβ42 and improve behavioral abnormalities (Hwang et al., 2019). Conversely, another study revealed a correlation between an increase in the plasma CTSB levels and the progression of MCI and AD from the mild to the severe forms of the disease, with the pathological worsening of the disease being mediated by protein accumulation (Morena et al., 2017). However, CTSB also possesses neuroprotective and anti-amyloidogenic properties (Mueller-Steiner et al., 2006), and mice studies have revealed neuronal loss and brain atrophy in double-KO mice lacking both CTSB and CTSL (Felbor et al., 2002). Additionally, CTSB may also be involved in the exercise-mediated enhancement of hippocampal neurogenesis, memory, and learning. However, further research is needed to determine the extent to which this myokine influences the exercise-mediated improvement in cognitive function in humans.

#### 4.3.6 LIF

LIF was first identified as a factor that promotes macrophage differentiation. It is produced by cardiac, neural, and skeletal muscles and acts as a versatile myokine (Hilton, 1992). LIF plays a role in controlling the proliferation and differentiation of satellite cells, which are crucial for muscle hypertrophy (Spangenburg and Booth, 2006). Studies have shown that LIF stimulates human myoblast proliferation and induces JunB and c-Myc expression in human myotubes. Conversely, the suppression of the LIF receptor has been shown to reduce myoblast proliferation (Broholm et al., 2011). LIF also has the ability to cross the blood‒brain barrier and enter the nervous system, where it plays a role in astrocyte development and oligodendrocyte survival. Treatment of mouse hippocampal cells with LIF has been found to activate the Akt/mTOR and STAT3 signaling pathways, downregulate LC3-II, increase c-fos expression (a marker of neuronal activation), and enhance cell survival. Similar results have also been observed in a *Drosophila* model of AD wherein LIF was found to inhibit LC3-II (Lee et al., 2019). Multiple studies have revealed elevated LIF levels in patients with AD, suggesting a possible connection between Aβ and the initiation of LIF-induced inflammation (Soilu-Hänninen et al., 2010). Theoretically, Aβ acts as a trigger for LIF, leading to an inflammatory response in individuals with AD. However, LIF also possesses neuroprotective properties, which it exerts by inducing oligodendrocyte apoptosis via TNF-α (Vanderlocht et al., 2006). In a drug trial involving rats with CNS injury, LIF was found to boost neurotrophin expression and facilitate corticospinal axon growth (Pan et al., 2000). However, owing to its potential instability, the impact of LIF on neural tissues was limited to a short duration. Further exploration is needed to address these limitations and establish LIF as a promising therapeutic option for the treatment of AD.

### 4.4 Optimal exercise prescription for myokine secretion and function

Previous research has confirmed that different types of physical activities affect skeletal muscle metabolic processes via unique mechanisms. Similarly, the secretion of myokines is also influenced by the specific nature of the physical activity (Little et al., 2018). The myokines produced during exercise have been shown to impact the muscle-brain communication surrounding musculoskeletal aging and MCI. Nevertheless, the secretion patterns of many other myokines remain somewhat unclear and are subject to various influences, including the intensity, type, and duration of exercise, as well as individual traits. It appears that exercise can trigger incremental changes ranging from minor to substantial, with peak levels observed immediately post-workout and within the first hour, followed by a return to baseline levels between 180 min and 24 h post-exercise (Bettariga et al., 2024). Both age and training status influence the myokine response to an acute bout of blood-flow restricted resistance exercise (Cordingley et al., 2023). Research on resistance exercise in individuals susceptible to probable MCI has revealed higher levels of IL-6 in blood markers than in controls do, particularly in older adults at high risk of MCI (Vints et al., 2024). A possible interpretation for the increase in IL-6 could be that resistance exercise was too intense for the participating older adults (Morishita et al., 2019). Additionally, sex appears to be a significant determinant of the IGF-1 response to exercise training. Research has indicated that older women exhibit variations in IGF-1 levels following elastic band resistance exercise (Hofmann et al., 2016), whereas younger men show increased IGF-1 levels in response to strength training programs (Takano et al., 2005). This discrepancy may be attributed to the fact that, with aging, persistent inflammation can inhibit the GH/IGF-1 axis (Schmidt et al., 2011). Additionally, while it is known that BDNF can traverse the blood-brain barrier, the release of myogenic BDNF into the bloodstream and its impact on the brain are still debatable (Rasmussen et al., 2009). Conversely, other myokines such as CTSB and irisin, which are regulated by exercise, are released into the bloodstream and cross the blood-brain barrier to increase the brain BDNF levels (Moon H. Y. et al., 2016; Momenzadeh et al., 2022). Maass et al. (2016) demonstrated that the inhibition of the IGF-1 receptor leads to the suppression of the exercise-induced upregulation of pro-BDNF and BDNF expression in the hippocampus, indicating that IGF-1, an upstream factor of BDNF, plays a role in hippocampal neurogenesis and BDNF gene regulation. In an 8-week study comparing cardiovascular and resistance exercise, resistance exercise alone was found to activate the IGF-1/Akt signaling pathway, enhancing hippocampal synaptic plasticity. This study revealed that the molecular mechanisms underlying hippocampal synaptic plasticity varied depending on the type of exercise (Cassilhas et al., 2012).

Different types of exercise may have different effects on the blood IGF-1 levels and facilitate the transport of IGF-1 from the bloodstream to the brain. For instance, a study that tracked IGF-1 levels overnight after intense resistance training revealed that the IGF-1 levels remained unaltered overnight (Nindl et al., 2001). In contrast, a lower-intensity resistance exercise routine may be more advantageous for older adults who prefer to avoid exerting greater effort and wish to mitigate potential injuries associated with heavier loads. Additionally, increasing the total load can be more easily achieved by increasing the number of repetitions at lower intensities (Cornish et al., 2018). It is crucial to consider the duration and intensity of exercise when aiming to increase blood IGF-1 levels, especially when dealing with resistance exercise, to prevent injuries in elderly individuals or patients. Although exercise is a recognized method for promoting good health and maintaining normal brain function and research shows that physical activity can have positive effects on human health and help individuals with conditions such as MCI and AD, caution is important, as high-impact and high-intensity exercise can have negative consequences. Studies indicate that engaging in low-intensity exercise, rather than high-intensity exercise, is more beneficial for protecting and revitalizing the aging brain in individuals with AD (Kim et al., 2003). It is important to consider the duration and intensity of exercise when aiming to increase blood IGF-1 levels, especially when dealing with resistance exercise, to prevent injuries in elderly individuals or patients. Although exercise is a recognized method for promoting good health and maintaining normal brain function and research shows that physical activity can have positive effects on human health and help individuals with conditions such as MCI and AD, caution is important, as high-impact and high-intensity exercise can have negative consequences. Studies indicate that engaging in low-intensity exercise, rather than high-intensity exercise, is more beneficial for protecting and revitalizing the aging brain in individuals with AD (Kim et al., 2003). Additionally, certain studies have indicated that cardiovascular and resistance training may have a more significant impact on cognitive function in individuals with MCI than multimodal exercise interventions do. Therefore, incorporating cardiovascular or resistance training into exercise regimens could serve as an effective strategy to mitigate cognitive decline in this population (Akalp et al., 2024). Consequently, implementing suitable exercise might orchestrate the interplay between the various myokines and provide health benefits.

Exercise, as an effective non-pharmacological intervention, can impact multiple organs of the body simultaneously. Different organs and tissues respond to exercise at the same time, highlighting the intricate network of mechanisms involved. While neuro-endocrine-immune network regulation is important, the communication between organs and tissues through exercise-induced myokines is also crucial. However, the relationship between physical activity parameters (such as type, intensity, and frequency) and the secretion of myokines remains unclear, impeding the development of targeted exercise programs. Future research should focus on understanding how physical activity influences the secretion of myokines and the effects of myokines on distant target organs. This knowledge will help establish connections between exercise, myokines, and chronic diseases, offering new insights into the health benefits of exercise. Moreover, it is essential to not only identify the role of individual myokines but also evaluate how different factors interact synergistically to impact the entire body. Furthermore, Various research studies have demonstrated the diverse nature of cognitive function changes with age, as some older individuals retain their cognitive abilities, while others experience mild or severe declines (Yaffe et al., 2009). Moving forward, artificial intelligence (AI) algorithms and biomarkers (D'Amico et al., 2020), such as physiological and myokines integrated into AI indices, hold promise in offering more precise and dependable approaches for identifying and addressing the biological markers of cognitive decline or sarcopenia across different age groups.

### 4.5 Limitation

This review examines the myokines that are known to play a significant role in the pathophysiology of musculoskeletal aging and mild cognitive impairment, aiming to inspire researchers to identify new strategies for developing drugs or interventions that mimic the health effects of exercise. Exploring the interactions between tissues and organs, one of the health benefits of exercise, along with their molecular mechanisms, will provide a solid foundation for creating personalized exercise prescriptions for musculoskeletal aging and mild cognitive impairment and is poised to become a leading focus in the field of exercise health studies. However, this review has certain limitations; the main limitation is that this is a narrative review rather than a complete systematic review. Nevertheless, this study represents the first attempt to investigate the relationships among myokines, aging-associated muscle atrophy, and cognitive dysfunction from an exercise perspective. There is a clear need for further research and a systematic review in this area. Only well-designed studies without methodological information will help expand our understanding of the connections between musculoskeletal aging, cognitive dysfunction due to aging, and their treatment implications.

## 5 Conclusion and outlook

The muscles and the brain, which are the primary organs responsible for locomotion, are interconnected systems that communicate with each other and share vital functions, including immunity and nutrition, in addition to locomotion. Their developmental processes are mutually regulated, with their crosstalk and the underlying mechanisms seen as complementary and reciprocal physiological mechanisms rather than independent functional regulations. Exercise acts as a mediator for the secretion of certain muscle-secreted factors, such as IL-6, IGF-1, BDNF, CTSB, irisin, and LIF, establishing a self-regulating loop between the brain and the muscles, thus enhancing the cognitive functions of the brain and the skeletal muscle functions.

Research on the regulation of skeletal muscle atrophy and mild cognitive impairment by myokines has gained significant attention in the fields of contemporary geriatrics and exercise physiology. Myokines may play an important role in the interplay between sarcopenia and cognitive function by regulating their pathogenesis and influencing clinical and pathological injuries. The identification of characteristic risk factors and markers for muscular atrophy in patients with mild cognitive impairment is a new direction. The myokines discussed here are only the tip of the iceberg, with some questions surrounding them requiring further clarification: the exact mechanisms underlying myokine release, the dose-effect relationship between exercise and exercise-induced myokine release, and the precise mechanisms underlying the regulation of the muscle-brain loop by myokines. Further research on the cross-talk in the muscle-brain axis will deepen the understanding of the mechanisms by which exercise promotes health and could pave the way for new avenues in exercise physiology research.
